# Risk of hospitalization in synucleinopathies and impact of psychosis

**DOI:** 10.3389/fnagi.2023.1274821

**Published:** 2023-09-22

**Authors:** Capucine Piat, Aidan F. Mullan, Cole D. Stang, Mania Hajeb, Emanuele Camerucci, Pierpaolo Turcano, Peter R. Martin, James H. Bower, Rodolfo Savica

**Affiliations:** ^1^Mayo Clinic Department of Neurology, Rochester, MN, United States; ^2^Mayo Clinic Department of Health Sciences Research, Rochester, MN, United States; ^3^Department of Neurology, Kansas University Medical Center, Kansas City, KS, United States

**Keywords:** synucleopathy, parkinsonism, psychosis, population-based, adverse event, hospitalization

## Abstract

**Background:**

Few studies have investigated the risk of hospitalization among patients with synucleinopathies (Parkinson disease, Dementia with Lewy Bodies, Parkinson disease dementia, Multiple System Atrophy) with associated psychosis and the impact of antipsychotic treatments on hospital admissions and duration of the stay.

**Objective:**

To determine the risk of hospitalization among patients with synucleinopathies and in patients with associated psychosis. To evaluate the impact of antipsychotic treatments on hospital admission of patients with synucleinopathies and psychosis in an incident cohort study in Olmsted County, Minnesota (MN).

**Methods:**

We used the Rochester Epidemiology Project (REP) to define an incident cohort of patients with clinically diagnosed synucleinopathies (1991–2010) in Olmsted County, MN. A movement disorder specialist reviewed all medical records to confirm the clinical diagnosis of synucleinopathies using the NINDS/NIMH unified diagnostic criteria.

**Results:**

We included 416 incident cases of clinically diagnosed synucleinopathies from 2,669 hospitalizations. 409 patients (98.3%) were admitted to the hospital at least once for any cause after the onset of parkinsonism. The median number of hospitalizations for a single patient was 5. In total, 195 (46.9%) patients met the criteria for psychosis: patients with psychosis had a 49% (HR = 1.49, *p* < 0.01) increased risk of hospitalization compared to patients without psychosis. Among patients with psychosis, 76 (39%) received antipsychotic medication. Treatment with antipsychotic medications did not affect the risk of hospitalization (HR = 0.93, *p* = 0.65). The median length of hospitalization among the entire cohort was 1 (IQR 0–4) day. There was no difference between hospitalization length for patients with no psychosis and patients with active psychosis (RR = 1.08, *p* = 0.43) or patients with resolved psychosis (RR = 0.79, *p* = 0.24).

**Conclusion:**

Psychosis increases the risk of hospitalization in patients with clinically defined synucleinopathies; however, it does not affect the length of hospital stays in our cohort. Antipsychotic treatment does not affect the risk of hospitalization in our study.

## Introduction

Parkinson’s disease-associated psychosis (PDP) affects 39%–60% of patients with Parkinson disease (PD) according to population-based studies. Psychosis also affects patients with other synucleinopathies, such as Lewy Body Disease (LBD), Parkinson’s disease with dementia (PDD), and Multiple System Atrophy (MSA) (Papapetropoulos et al., 2007; Badwal et al., 2022). Previous studies have linked PDP with an increased risk of mortality (Forsaa et al., 2010; Stang et al., 2022) as well as a greater caregiver burden (Aarsland et al., 1999; Schrag et al., 2006). PD patients with psychosis have an increased risk of nursing home and hospital admission (Goetz and Stebbins, 1993; Goetz and Stebbins, 1995; Wetmore et al., 2019). Although drugs such as pimavanserin and clozapine are available to treat psychosis, off-label use of antipsychotic medication is still common in PDP treatment, worsening motor symptoms for some of these drugs (Schneider et al., 2017).

Our study aims to determine the risk of hospitalization among synucleinopathies patients with or without psychosis and evaluate the impact of antipsychotic treatment on hospital admission of patients with psychosis.

## Methods

### Database and cohort design

We collected data using the electronic medical records linkage system of the Rochester Epidemiology Project (REP). The REP encompasses all local medical facilities, including private practitioners and nursing homes within Olmsted County, Minnesota (MN) with a catchment rate of over 97% in this population (St Sauver et al., 2011). The electronic index of the REP includes records, diagnostic and procedure codes, health services utilization data, outpatient drug prescriptions, demographics, and additional medical data (St Sauver et al., 2011).

Our incident cohort 1991–2010 was composed of two subcohorts: the first one was population-based and included all synucleinopathies cases in Olmsted County (1991–2005); the second one was composed of patients with synucleopathies with a parkinsonism onset within the 1991–2005 time period, who were diagnosed between 2006 and 2010. The purpose of the latter subcohort is to ensure catchment of individuals with parkinsonian motor symptoms onset in the 1991–2005 timeframe but with diagnosis within the next 5 years (2006–2010); this methodology has been already reported elsewhere (Savica et al., 2013) and its rationale is to maximize true population-based results for the first cohort (1991–2005).

All hospitalizations that occurred following the parkinsonism motor-symptom onset were recorded and categorized based on the reason for hospital admission, based on physicians’ history and physical examinations (H&Ps) and on a “principal problem list” as reported in the patients’ clinical records. All other contributing causes of hospital admission were also recorded, based on an “active hospital problems” list in patients’ clinical records, allowing for each hospitalization to have multiple categories. Hospitalizations were categorized in three time periods relative to the onset of psychosis: non-psychosis hospitalizations, psychosis hospitalizations, and hospitalizations after the resolution of psychosis. All recorded hospitalizations were considered non-psychosis hospitalizations in patients who never developed psychosis.

### Case ascertainment

We ascertained cases of synucleinopathies through the REP infrastructure, in a similar fashion to what previously reported (Savica et al., 2013). A movement-disorders specialist (RS) reviewed the medical records of patients with synucleinopathies to confirm their clinical diagnosis. A board-certified neuropathologist reviewed available autopsy reports to confirm the diagnosis when possible. Further details regarding the clinicopathologic characteristics of this cohort appear elsewhere; however, the clinical-pathology concordance was 89.5% of the cases (Turcano et al., 2017).

### Diagnosis of synucleinopathies

Synucleinopathies include Parkinson’s Disease (PD), Lewy Body disease (LBD), Parkinson’s disease with dementia (PDD), and Multiple System Atrophy (MSA). All our patients with synucleinopathies had parkinsonism, and some had additional distinct clinical features relative to the specific clinical syndrome. Parkinsonism is the presence of at least two of four cardinal signs: rest tremor, bradykinesia, rigidity, and impaired postural reflexes. These criteria have proven efficient in a previous study (Savica et al., 2013) where a movement-disorder neurologist blinded to the criteria reviewed a sample of patients with parkinsonism. The agreement on the presence of parkinsonism was 90%, higher than the accuracy of the UK Brain Bank Criteria on parkinsonism (82%) (Hughes et al., 1992). Fourth Consensus criteria and McKeith criteria for Dementia with Lewy Bodies (DLB)/PDD (McKeith et al., 2017) were adopted (Gilman et al., 1998, 2008) for MSA.

### Identification of patients with psychosis

We used the NINDS/NIMH Work Group clinical criteria for psychosis to classify patients with psychosis (Ravina et al., 2007). We defined psychosis as the presence of one or more of the following symptoms: illusions, false sense of presence, hallucinations, or delusions. Psychotic symptoms had to last at least 1 month or recur within 1 month and not be explained by another medical condition (Ravina et al., 2007). We excluded all cases of acute change in mental status such as acute dementia, confusion, and delirium due to medical conditions or adverse drug events. If the symptoms persisted for 6 months or more, patients were considered to have unresolved psychosis. Patients with psychosis were regarded as treated if any antipsychotic prescription was used for psychosis at any time after their psychosis diagnosis. Available antipsychotic medications at the time of the study were Quetiapine, Clozapine, Olanzapine, Risperidone, and Haloperidol.

### Statistical analysis

Numeric features were summarized with medians and interquartile ranges; categorical features were summarized with frequency counts and percentages. The incidence of hospitalizations among synucleinopathy patients was calculated as the total number of hospitalizations divided by the total years of follow-up recorded for the entire cohort. Comparisons between males and females were conducted using incidence rate ratios (IRRs) with 95% confidence intervals.

Risk of hospitalization was assessed using recurrent-event Andersen-Gill (AG) models. Psychosis was treated as a time-dependent covariate to properly account for the differential onset and resolution of psychosis within the cohort of synucleinopathy patients. The AG models also accounted for patient sex and age at the onset of parkinsonism. A secondary analysis further classified psychosis as either treated with antipsychotic medication or untreated. This predictor was again treated as a time-dependent covariate since patients could have different lengths of untreated and treated psychosis depending on when the medication was prescribed relative to the onset of psychosis.

The length of hospitalization was compared between psychosis time periods using population-averaged Poisson generalized estimating equations (GEEs). The strength of these models over simple regression is that GEEs account for multiple hospitalizations from a single patient which will not necessarily be independent. Models were unadjusted using psychosis as the predictor of interest, as well as adjusted for patient sex and age at parkinsonism onset. Model results are reported as rate ratios (RRs) with 95% confidence intervals.

### Standard protocol approvals, registrations, and patient consents

The Mayo Clinic and Olmsted Medical Center Institutional Review Boards approved this study. Participating patients or their legally authorized representatives provided informed written consent for the use of their medical information for research (Savica et al., 2013).

## Data availability statement

All the relevant data are shared and published in this article. Data regarding case ascertainment and methodology on case identification have been previously published (Savica et al., 2013; Stang et al., 2022). Data regarding frequency of psychosis, mortality, and other characteristics of patients of this cohort have already been reported elsewhere (Stang et al., 2022).

## Results

### Demographics

A total of 416 clinically diagnosed synucleinopathy patients were included in this study. Table 1 provides a summary of patients’ demographics, parkinsonism characteristics, and hospitalizations. Our cohort was 37.2% female and 63.8% male. The median age of onset was 75 years (IQR = 67–81). The median time of follow-up was 8.5 years (5–12.1). Breaking down by synucleopathy type, 283 (68%) of our patients had Parkinson’s disease, 76 (18.3%) Lewy Body Disease, 46 (11.1%) Parkinson’s with dementia, and 11 (2.6%) Multiple System Atrophy.

**Table 1:** Summary of cohort demographics and disease characteristics.

	Female (*N* = 155)	Male (*N* = 261)	All patients (*N* = 416)
**Demographics**			
**Age at onset**, years	76 (68, 82)	75 (67, 80)	75 (67, 81)
**Race**, *n* (%)			
White	150 (96.8%)	250 (95.8%)	400 (96.2%)
Non-White	4 (2.6%)	8 (3.1%)	12 (2.9%)
Unknown	1 (0.6%)	3 (1.1%)	4 (1.0%)
**Disease characteristics**			
**Parkinsonism subtype**, *n* (%)			
Parkinson’s disease	110 (71.0%)	173 (66.3%)	283 (68.0%)
Lewy body disease	22 (14.2%)	54 (20.7%)	76 (18.3%)
Parkinson’s disease with dementia	21 (13.5%)	25 (9.6%)	46 (11.1%)
Multiple system atrophy	2 (1.3%)	9 (3.4%)	11 (2.6%)
**Duration of follow-up**, years	9.2 (4.7, 12.7)	8.2 (5.2, 11.6)	8.5 (5.0, 12.1)
**Psychosis,** *n* (%)	71 (45.8%)	124 (47.5%)	195 (46.9%)
**Hospitalizations**			
**Total hospitalizations**, median (Q1, Q3)	5 (2, 9)	5 (3, 8)	5 (2, 9)
**Any hospitalization**, *n* (%)	153 (98.7%)	256 (98.1%)	409 (98.3%)
Neurological	114 (73.5%)	189 (72.4%)	303 (72.8%)
Cardiovascular	102 (65.8%)	182 (69.7%)	284 (68.3%)
Infection	75 (48.4%)	115 (44.1%)	190 (45.7%)
Respiratory	58 (37.4%)	109 (41.8%)	167 (40.1%)
Psychiatric	73 (47.1%)	93 (35.6%)	166 (39.9%)
Nephrological	49 (31.6%)	109 (41.8%)	158 (38.0%)
Fall	61 (39.4%)	80 (30.7%)	141 (33.9%)
Trauma	43 (27.7%)	64 (24.5%)	107 (25.7%)
Other	136 (87.7%)	230 (88.1%)	366 (88.0%)

### Incidence of hospitalization

The 416 patients included in the cohort had a total of 2,669 hospitalizations. Most patients (409/416, 98.3%) were admitted to the hospital at least once after parkinsonism onset and the rate of admission was similar between males and females (*p* = 0.98).

In the full cohort of synucleinopathy patients, the incidence of hospitalizations following the onset of parkinsonism was 70.6 hospitalizations per 100 person-years of follow-up. The incidence of hospitalization was similar between males (70.5 per 100 person-years) and females (70.6 per person-years; IRR = 1.00, 95% CI: 0.93–1.08, *p* = 0.99).

### Psychosis

Of the 416 synucleinopathy patients, 195 (46.9%) developed psychosis. Table 2 provides a summary of patients’ parkinsonism characteristics, psychosis characteristics, treatment, and hospitalizations. This subgroup consisted of 124 (47.5%) males and 71 (45.8%) females. The median age at parkinsonism onset and at psychosis onset was 74 years (68–81) and 79 years (74–85) respectively. The median time of follow-up time to psychosis onset was 9.1 years (5.7–12.7). Seventy-six of the 195 (39%) patients with psychosis were treated with antipsychotics. One hundred and thirty eight of 195 (70.8%) had unresolved psychosis.

**Table 2:** Summary of patients diagnosed with psychosis.

	Female (*N* = 71)	Male (*N* = 124)	All patients (*N* = 195)
**Parkinsonism characteristics**			
**Age at parkinsonism onset**, years	74 (65, 81)	74 (69, 80)	74 (68, 81)
**Parkinsonism subtype**, *n* (%)			
Parkinson’s disease	48 (67.6%)	66 (53.2%)	114 (58.5%)
Lewy body disease	16 (22.5%)	44 (35.5%)	60 (30.8%)
Parkinson’s disease with dementia	7 (9.9%)	12 (9.7%)	19 (9.7%)
Multiple system atrophy	0 (0%)	2 (1.6%)	2 (1.0%)
**Duration of follow-up**, years	10.2 (6.6, 14.3)	8.6 (5.6, 11.2)	9.1 (5.7, 12.7)
**Psychosis characteristics**			
**Age at psychosis onset,** years	79 (73, 86)	79 (74, 84)	79 (74, 85)
**Psychosis symptoms,** *n* (%)			
Hallucinations	62 (87.3%)	113 (91.1%)	175 (89.7%)
Delusions	22 (31.0%)	40 (32.3%)	62 (31.8%)
Confusion	29 (40.8%)	40 (32.3%)	69 (35.4%)
Delirium	14 (19.7%)	14 (11.3%)	28 (14.4%)
**Treatment for psychosis**, *n* (%)			
Any antipsychotic medication	32 (45.1%)	44 (35.5%)	76 (39.0%)
Quetiapine	31 (43.7%)	43 (34.7%)	74 (37.9%)
Haloperidol	3 (4.2%)	7 (5.6%)	10 (5.1%)
Risperidone	4 (5.6%)	0 (0%)	4 (2.1%)
**Hospital admission due to psychosis,** *n* (%)	5 (7.0%)	25 (20.2%)	30 (15.4%)
**Psychosis resolution**, *n* (%)			
Unresolved	47 (66.2%)	91 (73.4%)	138 (70.8%)
Resolved	24 (33.8%)	33 (26.6%)	57 (29.2%)

### Risk of hospitalization

In an unadjusted AG model including only psychosis as a predictor of hospitalization, we observed that active psychosis correlated with a 49% greater risk for hospitalization (HR = 1.49, 95% CI: 1.25–1.79, *p* < 0.001). However, the risk of hospitalization for patients with resolved psychosis did not differ significantly from patients without a diagnosis of psychosis (HR = 1.07, 95% CI: 0.74–1.53, *p* = 0.72).

After accounting for patient age, sex, and synucleinopathy subtype, we found comparable results. Active psychosis correlated with a 41% increase in risk for hospitalization (HR = 1.41, 95% CI: 1.17–1.70, *p* < 0.001). Resolved psychosis was not significantly different from no psychosis (HR = 0.93, 95% CI: 0.63–1.37) and was associated with a 34% lower risk of hospitalization compared to active psychosis (HR = 0.66, 95% CI: 0.44–0.98, *p* = 0.042).

There was no difference in risk of hospitalization between males and females (HR = 1.04, 95% CI: 0.87–1.23, *p* = 0.69); however, a 5-year increase in age was associated with a 16% increase in risk for hospitalization (HR = 1.16, 95% CI: 1.14–1.18, p < 0.001).

### Effect of anti-psychotic treatment on risk of hospitalization

When classifying psychosis as treated by antipsychotic medication or untreated, an unadjusted AG model found both treated (HR = 1.46, 95% CI: 1.21–1.77, p < 0.001) and untreated psychosis (HR = 1.58, 95% CI: 1.16–2.15, *p* = 0.004) associated with greater risk for hospitalization. There was no significant difference between treated and untreated psychosis (HR = 0.93, 95% CI: 0.67–1.29, *p* = 0.65).

Similarly, after accounting for patient age, sex, and synucleinopathy type, both treated (HR = 1.38, 95% CI: 1.14–1.69, *p* = 0.001) and untreated psychosis (HR = 1.47, 95% CI: 1.07–2.03, *p* = 0.018) were associated with greater risk of hospitalization. There was no difference between the two categories of psychosis on risk of hospitalization (HR = 0.94, 95% CI: 0.67–1.32, *p* = 0.72).

### Duration of hospitalization

The median length of hospitalization among the entire cohort was 1 (IQR 0–4) day. For males the median was 1 (0–4) day and for females the median was 1 (0–4) day of hospitalization. An unadjusted GEE model found no difference in length of hospitalization between males and females (RR = 0.96, 95% CI: 0.78–1.20, *p* = 0.74).

Hospitalizations for patients not previously diagnosed with psychosis lasted a median of 1 (IQR 0–4) day. Comparatively, patients with active psychosis had a median of 2 (IQR 0–5) days and patients with resolved psychosis had a median of 1 (0–3) day of hospitalization. Relative to the patients not diagnosed with psychosis, there was no difference in hospitalization length for patients with active psychosis (RR = 1.08, 95% CI: 0.89–1.32, *p* = 0.43) or patients with resolved psychosis (RR = 0.79, 95% CI: 0.52–1.18, *p* = 0.24). There was also no difference between patients with resolved psychosis and patients with active psychosis (RR = 0.73, 95% CI: 0.49–1.07, *p* = 0.10).

Adjusting for patient age and sex revealed no difference in length of hospitalization for patients with active psychosis (RR = 1.12, 95% CI: 0.91–1.37, *p* = 0.29) or resolved psychosis (RR = 0.81, 95% CI: 0.55–1.19, p = 0.29). There was no difference between patients with resolved psychosis and patients with active psychosis (RR = 0.73, 95% CI: 0.50–1.06, *p* = 0.095). Similarly, males did not have significantly different hospitalization duration compared to females (RR = 0.97, 95% CI: 0.77–1.22, *p* = 0.76).

### Reasons for hospitalization

The most frequent cause of hospitalization was neurological, with 72.8% of all patients hospitalized at least once for neurological reasons. The least common cause was trauma, with 25.7% of all patients having at least one trauma-related hospitalization.

## Discussion

### Risk of hospitalization

Our study shows that active psychosis increased by 49% the risk of hospitalization of patients with synucleinopathies. This association was significant after accounting for patients’ age, sex, and parkinsonism subtype, with a 41% increased relative risk of hospitalization. Resolved psychosis mitigated this higher risk, and patients had the same hospitalization risk as patients without psychosis.

### Effect of antipsychotic treatment on hospitalization

In this study, we found that antipsychotic treatment by Quetiapine, Olanzapine, Risperidone, and Haloperidol did not significantly affect the risk of hospitalization compared to patients with untreated psychosis. Similarly, we found that antipsychotic treatment did not significantly affect hospitalization length. Although a relatively small number of our patients with psychosis were treated [76 of 195 (39%)], and none of our patients received Clozapine, this suggests that the other drugs available at the time of our study (1991–2010) may help reduce psychotic symptom burden for both patients and caregivers but are not necessarily effective for reducing the risk or length of hospitalization in patients with synucleinopathies. The findings support a previously published study (Stang et al., 2022), where antipsychotic treatment available at that time did not significantly affect mortality of PD patients with psychosis, even if they helped reduce psychotic symptoms. It is important to note that since our study time-period, other drugs such as pimavanserin have become available, and the use of clozapine is also possible, although rarely used.

### Duration of hospitalization

In our study, the median length of hospitalization stay was relatively short [1 (IQR 0–4) day], compared to a study on PD patients from the United Kingdom (Woodford and Walker, 2005) where the median length of hospital stay was 21 days, and another study on Spanish PD patients (Gil-Prieto et al., 2016), where the median length of hospital stay was 10 days. A possible contributing factor of these different hospitalization lengths could be that our study includes Emergency Department admissions, with a vast number of patients discharged the next day.

An interesting result from our study is that the presence of active psychosis does not affect the length of hospitalization, compared to patients with no psychosis or resolved psychosis. An explanation for this finding may be the effort made by healthcare teams to limit hospitalization to the shortest possible duration for parkinsonian patients to reduce the risk of adverse events or higher mortality (Low et al., 2015). Another explanation is that resolution of the acute phase of psychosis may bring patients back to their baseline (even with persistent psychotic features), and a longer hospitalization may not be necessary. As hospitalization itself can cause delirium for PD patients (Ahlskog, 2014), short as possible hospitalizations still offer the safest approach for parkinsonian patients, except when patients are a danger to themselves or others.

### Rate of hospitalization

In our study, the median number of hospitalizations for a single patient was 5 (IQR 2–9). This is higher than previously reported (Okunoye et al., 2022).

### Reasons for hospitalization

The most common reasons for hospitalization in our cohort were neurological (72.8%) and cardiovascular (68.3%), followed by infections (45.7%). Falls only accounted for 25.7% of hospitalizations. Interestingly, this is in contrast with multiple previous studies on parkinsonism-related hospitalizations where the leading cause of hospitalizations were falls (Hommel et al., 2022; Okunoye et al., 2022; Santos Garcia et al., 2023).

### Limitations and strengths

Our study has limitations. First, retrospective data collection is dependent on physician report of symptoms, which can lead to an underascertainment of psychosis. Second, as psychosis rates vary widely in synucleinopathy patients (Forsaa et al., 2010; Stang et al., 2022), and as not all patients with psychosis need medication, the number of treated patients with psychosis was relatively small. This can affect the generalizability of the results. Third, data on antipsychotic treatment adherence were not available so there is risk of bias. Fourth, pimavanserin, a drug proven efficient on PDP (and psychosis-related hospitalizations) was not available in our study time-period (Rajagopalan et al., 2023a,b). The current study can be relevant as a baseline to set the stage for future population-based studies that might confirm our results with readily available pimavanserin. Fifth, although clozapine was available at the time of our study, none of our patients received this medication, which may affect the generalizability of our results. Finally, we had a limited number of pathologically confirmed cases (24 cases) in our cohort. However, a previous study shows that our rate of clinical diagnosis accuracy is 89.5% (Turcano et al., 2017).

Our study also has several strengths. First, the complete review of medical records by a neurologist, to confirm both diagnosis of synucleinopathy and psychosis, insures the good quality of our data and less selection bias seen in studies based on electronic codes only (Peterson et al., 2020). Second, our study is population-based, with our cohort grouping all incident cases of synucleinopathy from 1991 to 2010 in Olmsted County, Minnesota. Third, this study is one of the first to look at hospitalization risk of all synucleinopathy patients and specifically target the influence of psychosis. This is of interest as psychosis is known to increase mortality in parkinsonian patients (Stang et al., 2022) and affect their quality of life (Schneider et al., 2017; Badwal et al., 2022).

## Conclusion

Our study shows that active psychosis increases the risk of hospitalization of synucleinopathies patients by 49% but does not affect the duration of hospitalization. Available antipsychotic treatment at the time of the study does not affect the risk of hospitalization. With nearly half (46.9%) of our patients with synucleinopathy affected by psychosis, it is necessary for health professionals, patients, and families to look out for psychosis symptoms throughout the disease course, to prevent deleterious consequences of psychosis on their life and autonomy.

## Data availability statement

The raw data supporting the conclusions of this article will be made available by the authors, without undue reservation.

## Ethics statement

The studies involving humans were approved by Mayo Clinic Institutional Review Board. The studies were conducted in accordance with the local legislation and institutional requirements. Written informed consent for participation in this study was provided by the participants’ legal guardians/next of kin.

## Author contributions

CP: Writing – original draft. AM: Investigation, Writing – review & editing, Formal analysis and Performed statistical analysis. CS: Data curation, Investigation, Writing – review & editing. MH: Investigation – review & editing. EC: Data curation, Investigation, Writing – review & editing, Methodology. PT: Data curation, Investigation, Methodology – review & editing. PM: Formal analysis – review & editing. JB: Conceptualization, Investigation, Writing – review & editing. RS: Conceptualization, Data curation, Formal analysis, Investigation, Methodology, Supervision, Writing – review & editing.

## Funding

The author(s) declare financial support was received for the research, authorship, and/or publication of this article. Rodolfo Savica receives research support from the National Institute on Aging, the National Institute of Neurological Disorders and Stroke, the Mayo Clinic Small Grants Program National Center for Advancing Translational Sciences (NCATS) and Acadia Pharmaceuticals Inc.

## Conflict of interest

The authors declare that the research was conducted in the absence of any commercial or financial relationships that could be construed as a potential conflict of interest.

The author(s) declared that they were an editorial board member of Frontiers, at the time of submission. This had no impact on the peer review process and the final decision.

## Publisher’s note

All claims expressed in this article are solely those of the authors and do not necessarily represent those of their affiliated organizations, or those of the publisher, the editors and the reviewers. Any product that may be evaluated in this article, or claim that may be made by its manufacturer, is not guaranteed or endorsed by the publisher.
